# The Impact of Medical School Psychiatry Exposure on Consideration of Psychiatry as a Career

**DOI:** 10.1192/bjo.2026.11333

**Published:** 2026-06-30

**Authors:** Ariela Carno, Amelia Leigh, Elle Borges Michel, Rabia Abdul Khaliq, Priya Nagalingam

**Affiliations:** Hertfordshire Partnership University NHS Foundation Trust, Hertfordshire, United Kingdom

## Abstract

**Aims:**

To determine whether exposure to a psychiatry specialty at medical school increases interest in pursuing psychiatry as a career.

Since 2017, the mental health workforce has seen a year-on-year expansion in numbers overall. Although between 2010 and 2023, the number of psychiatrists increased by 22% this was significantly lower compared to a 43% increase for doctors in the NHS overall. Recruitment into psychiatric specialties remains a key challenge with many psychiatric specialties facing under-recruitment year on year. According to ‘Stepping forward to 2020/21: The mental health workforce plan for England’ there are 5400 Consultant Psychiatrists with a vacancy rate of 13%.

Hertfordshire Partnership Foundation NHS Trust (HPFT) provide psychiatric placement for Cambridge medical students (Year 5). During a Quality Assurance cycle in 2025, HPFT was rated the best from the 5 Mental Health Trusts that provide psychiatry placement. Despite thisbetween 2014 and 2016 only 1.6% of graduates from the University of Cambridge pursued Psychiatry as a clinical speciality following graduation compared to 4.3% from Keele Medical school graduates.

**Methods:**

Medical students attending a clinical placement in mental health between October 2024 and November 2025 were invited to complete a pre-placement and post-placement questionnaire that asked their likelihood of pursing Psychiatry for their foundation and/or core training choices. The questionnaire consisted of two questions that asked respondents to rate how likely they are in considering undertaking a psychiatry placement as part of their foundation/CT training on a Likert scale of Very likely, Likely, Neutral, Unlikely and Very Unlikely.

**Results:**

A total of 74 pre-placement and 77 post-placement questionnaire responses were received.

For FY choices: A 33% increase was observed in medical students very likely or likely to choose psychiatry as a foundation year option between pre-and post-placement.

For Core psychiatry training: A 20% increase was observed in medical students very likely or likely to choose psychiatry as a core trainee option between pre-and post-placement.

**Conclusion:**

Medical students’ experiences during a clinical placement in Psychiatry appear to contribute to the consideration of a career in psychiatry.

